# Inhibition of superoxide and iNOS augment cutaneous nitric oxide‐dependent vasodilation in non‐Hispanic black young adults

**DOI:** 10.14814/phy2.16021

**Published:** 2024-04-19

**Authors:** Brett J. Wong, Casey G. Turner, Matthew J. Hayat, Jeffrey S. Otis, Arshed A. Quyyumi

**Affiliations:** ^1^ Department of Kinesiology & Health Georgia State University Atlanta Georgia USA; ^2^ Molecular Cardiology Research Institute Tufts Medical Center Boston Massachusetts USA; ^3^ Department of Population Health Sciences, School of Public Health Georgia State University Atlanta Georgia USA; ^4^ Emory Clinical Cardiology Research Institute Emory University School of Medicine Atlanta Georgia USA

**Keywords:** endothelium, microdialysis, microvascular, oxidative stress

## Abstract

We assessed the combined effect of superoxide and iNOS inhibition on microvascular function in non‐Hispanic Black and non‐Hispanic White participants (*n* = 15 per group). Participants were instrumented with four microdialysis fibers: (1) lactated Ringer's (control), (2) 10 μM tempol (superoxide inhibition), (3) 0.1 mM 1400 W (iNOS inhibition), (4) tempol + 1400 W. Cutaneous vasodilation was induced via local heating and NO‐dependent vasodilation was quantified. At control sites, NO‐dependent vasodilation was lower in non‐Hispanic Black (45 ± 9% NO) relative to non‐Hispanic White (79 ± 9% NO; *p* < 0.01; effect size, *d* = 3.78) participants. Tempol (62 ± 16% NO), 1400 W (78 ± 12% NO) and tempol +1400 W (80 ± 13% NO) increased NO‐dependent vasodilation in non‐Hispanic Black participants relative to control sites (all *p* < 0.01; *d* = 1.22, 3.05, 3.03, respectively). The effect of 1400 W (*p* = 0.04, *d* = 1.11) and tempol +1400 W (*p* = 0.03, *d* = 1.22) was greater than tempol in non‐Hispanic Black participants. There was no difference between non‐Hispanic Black and non‐Hispanic White participants at 1400 W or tempol + 1400 W sites. These data suggest iNOS has a greater effect on NO‐dependent vasodilation than superoxide in non‐Hispanic Black participants.

## 1 INTRODUCTION

Rates of cardiovascular disease (CVD) and risk factors for CVD are higher in non‐Hispanic Black individuals relative to age‐matched non‐Hispanic White individuals (Tsao et al., 2023). While the pathogenesis of CVD and its risk factors is undoubtedly multifaceted, a substantial line of evidence indicates there is impaired endothelial‐dependent vasodilation of both the macro‐ and microvasculature in non‐Hispanic Black individuals relative to non‐Hispanic White individuals in both health and disease (Akins et al., 2022; Campia et al., 2002; Cardillo et al., 1998; Hurr et al., 2018; Kim et al., 2018; Kim & Brothers, 2020; Miller et al., 2021; Morris et al., 2012; Neuman et al., 2016; Ozkor et al., 2011, 2014, 2015; Patik et al., 2018; Shen et al., 2015; Turner et al., 2020; Turner, Hayat, et al., 2023; Wolf et al., 2020; Wong et al., 2020).

Endothelial function, typically defined as the magnitude of nitric oxide (NO)‐dependent vasodilation, is a non‐traditional risk factor for CVD. There is strong data showing an inverse relationship between NO‐dependent vasodilation and CVD, with an approximately 10% reduced risk of developing CVD for every 1% increase in NO‐dependent vasodilation (measured as flow‐mediated dilation of the brachial artery) (Green et al., 2011). Thus, assessment of endothelial function, particularly NO‐dependent vasodilation, can provide important insights into not only vascular health but also potential risk of developing CVD.

Assessment of microvascular endothelial function has important clinical implications since the microvasculature is the site of blood pressure regulation (via peripheral resistance) and glucose uptake. The cutaneous microvasculature is an ideal representative microvascular bed in that it is easily accessible, which allows for minimally‐invasive mechanistic studies, and correlates well with dysfunction in other less accessible microvascular beds, such as the renal circulation (Coulon et al., 2012; Holowatz et al., 2008; Kruger et al., 2006; Minson, 2010). Local heating of the skin results in a biphasic vasodilation of the cutaneous microvasculature. The initial vasodilation is mediated largely by sensory nerve‐dependent mechanisms (Minson et al., 2001; Wong & Fieger, 2010; Wong & Minson, 2011) whereas the second phase is mediated largely by endothelial‐dependent mechanisms (Bruning et al., 2012; Brunt & Minson, 2012; Choi et al., 2014; Fieger & Wong, 2010; Kellogg Jr., et al., 1999; Kellogg Jr. et al., 2008, 2009; Minson et al., 2001). Since eNOS‐derived NO is cardioprotective and the cutaneous vascular response is ~60%–80% dependent on eNOS, this represents an ideal stimulus to assess microvascular endothelial function. Data from our lab, and others, have shown reduced cutaneous vascular responses to local heating in non‐Hispanic Black young adults (Akins et al., 2022; Hurr et al., 2018; Kim et al., 2018; Kim & Brothers, 2020; Miller et al., 2021; Patik et al., 2018; Turner et al., 2020; Turner, Hayat, et al., 2023; Wolf et al., 2020; Wong et al., 2020). We recently demonstrated this reduction is due, in part, to activity of inducible NO synthase (iNOS), which is upregulated under stress conditions, such as increased levels of superoxide, and has been shown to contribute to reduced cutaneous NO‐dependent vasodilation in hypertensive humans (Massey et al., 2023; Miller et al., 2021; Putra et al., 2020; Smith et al., 2011; Tsai & Lein, 2021). Previous data also indicates inhibition of superoxide and oxidative stress can improve NO‐dependent vasodilation in non‐Hispanic Black young adults (Hurr et al., 2018; Kim & Brothers, 2020; Patik et al., 2018; Turner, Hayat, et al., 2023; Wolf et al., 2020). Data in animal models suggests the iNOS inhibitor 1400 W may also attenuate oxidative stress, nitrosative stress, and proinflammatory cytokines in addition to its direct effects on iNOS (Massey et al., 2023; Putra et al., 2020). Although there is a close relationship between superoxide and iNOS activity, superoxide represents only one specific form of oxidative stress and other reactive oxygen species could also increase activity of iNOS. Thus, combined inhibition of superoxide (tempol) and iNOS (1400 W) may yield greater improvements in microvascular function due to the pleiotropic nature of 1400 W. To our knowledge, there are no data in humans that have investigated the combined effect of superoxide and iNOS inhibition. Therefore, the purpose of this study was to test the hypothesis that, in non‐Hispanic Black young adults, concomitant inhibition of superoxide and iNOS would result in greater improvements in NO‐dependent cutaneous vasodilation than the independent effects of inhibiting superoxide and iNOS.

## 2 METHODS

### 2.1 Ethical approval

All experimental procedures were initially approved by Advarra Institutional Review Board (Columbia, MD; Pro00024265) and accepted by the Georgia State University Institutional Review Board, and approval for the use of all pharmacological substances was provided by the U.S. Food and Drug Administration (IND 138231). This study conformed to the guidelines set forth by the Declaration of Helsinki. All participants provided written and verbal informed consent prior to participating in any experimental procedure.

### 2.2 Participants

Participant demographics, hemodynamics, and blood variables are shown in Table 1. We recruited a total of 30 participants for this study: 15 who self‐identified as non‐Hispanic Black and 15 who self‐identified as non‐Hispanic White. All participants were healthy as determined from a self‐report health history with no diagnosis of any form of cardiovascular, respiratory, or metabolic disease. A total of 14 women participated in this study: Six non‐Hispanic Black women and eight non‐Hispanic White women. We did not control for menstrual cycle phase, oral contraceptive phase, or type of contraceptive but did record this information (Table 2). Four women declined to disclose information regarding their cycle phase and/or contraceptive due to privacy concerns. Participants who had an active COVID‐19 infection (or any other active respiratory illness) or had a known COVID‐19 infection within the past 1 month were excluded from the study (Dillon et al., 2022). Of the 30 participants, only three had a known positive COVID‐19 infection (one non‐Hispanic Black woman, two non‐Hispanic White men), all three had a positive test result more than 8 months prior to the study (range: 9–22 months), and all three reported having mild symptoms during COVID‐19 infection (slight fever, headache, cough).

**TABLE 1:** Participant demographics, hemodynamics, and blood variables.

	non‐Hispanic black	non‐Hispanic white
Demographics		
Women/Men, *n*	6/9	8/7
Age, years	22 ± 2	23 ± 6
Height, m	1.7 ± 0.1	1.8 ± 0.2
Mass, kg	73.4 ± 13.6	77.7 ± 13.0
BMI, kg/m^2^	23.7 ± 4.9	22.6 ± 2.4
Hemodynamics		
SBP, mmHg	111 ± 6	113 ± 5
DBP, mmHg	71 ± 4	71 ± 4
MAP, mmHg	84 ± 4	85 ± 4
HR, beats/min	67 ± 9	68 ± 7
Blood Variables		
HDL, mg/dL	51 ± 13	48 ± 17
LDL, mg/dL	94 ± 16	102 ± 20
Total Cholesterol, mg/dL	152 ± 23	146 ± 21
Glucose, mg/dL	84 ± 13	90 ± 12

*Note*: Values are mean ± SD.

Abbreviations: BMI, body mass index; DBP, diastolic blood pressure; HR, heart rate; HDL, high density lipoproteins; LDL, low density lipoproteins; MAP, mean arterial pressure; SBP, systolic blood pressure.

**TABLE 2:** Menstrual cycle and contraceptive information.

	non‐Hispanic black (*n* = 6)	non‐Hispanic white (*n* = 8)
Natural menstrual cycle phase, *n*		
Early Follicular (days 1–5)	3	2
Oral contraceptive pill phase & type, *n*		
Placebo	2	1
Active pill		2
Third generation		2
Fourth generation	2	1
Declined to answer	1	3
Total	6	8

*Note*: Values are number (*n*) of women with either a natural menstrual cycle or using oral contraceptive pills. Phase of menstrual cycle or contraceptive pill use is when each participant volunteered for the study. Oral contraceptive pill generation are they formulation used by each participant. The declined *to answer* row shows the number of women who chose not to disclose this information.

### 2.3 Instrumentation

Participants were seated in a semi‐recumbent position for the duration of the experiment with the experimental (left) arm at heart level to minimize the effect of hydrostatic pressure on blood flow. All participants had a fasting venous blood sample taken from a finger stick for analysis of blood lipids and glucose (Cholestech LDX, Abbott, Orlando, FL; Table 1). Participants were then instrumented with four intradermal microdialysis fibers (Custom CMA 31 probes with a 6 KDa molecular weight membrane; CMA Microdialysis, Harvard Apparatus, Hollister, MA) on the left dorsal forearm as described previously (Miller et al., 2021; Turner et al., 2020; Turner, Hayat, et al., 2023; Turner, Stanhewicz, et al., 2023; Wong et al., 2020). Microdialysis sites were assigned as: (1) lactated Ringer's (Baxter Healthcare, Deerfield, IL) to serve as a control, (2) 10 μM tempol (Sigma Aldrich, St. Louis, MO) to inhibit superoxide (Fujii et al., 2014), (3) 0.1 mM 1400 W (AdipoGen, San Diego, CA) to inhibit iNOS (Smith et al., 2011), and (4) combined tempol +1400 W (final concentrations of 10 μM and 1 mM, respectively). Drugs were diluted in sterile lactated Ringer's solution and drawn through sterile syringe filters (Acrodisc, 13 mm disk, 0.2 μm pore, hydrophilic PES membrane, USP class VI; Pall Corporation, Port Washington, NY). All drugs were infused for at least 30 min prior to the local heating protocol. Drugs were infused at a rate of 2 μL/min using syringe pumps (Beehive Controller and Baby Bee Pumps, Bioanalytical Systems, West Lafayette, IN).

Skin blood flow was measured at each microdialysis site (left dorsal forearm) using 7‐array integrated laser‐Doppler probes (VP7b probes and VMS‐LDF2 monitor, Moor Instruments; Axminster, UK). Local skin temperature at each microdialysis site was controlled with a local heating unit (VHP1 Heater Units and VMS‐HEAT controller; Moor Instruments; Axminster, UK), which also housed each laser‐Doppler probe.

An automated blood pressure cuff (Vital Signs Series 6000, Welch‐Allyn, Skaneateles Falls, NY) was placed on the right arm of each participant and blood pressure was measured every 5–8 min. Mean arterial pressure was calculated as 1/3 pulse pressure plus diastolic pressure.

### 2.4 Experimental protocol

Following trauma resolution associated with placement of microdialysis fibers (at least 30 min and up to ~60 min), baseline data was collected for 8–10 min with the local heaters set at 33°C. A standardized local heating protocol was initiated to elicit cutaneous vasodilation. Local heaters at each microdialysis site were increased from 33°C to 39°C at a rate of 0.1°C per second (Choi et al., 2014; Miller et al., 2021; Turner, Hayat, et al., 2023; Turner, Stanhewicz, et al., 2023; Wong et al., 2020). Heaters were held at 39°C until a plateau in skin blood flow was achieved (approximately 30–40 min of heating) and then 20 mM L‐NAME (nonspecific NO synthase inhibitor) was infused through each microdialysis fiber to quantify NO‐dependent vasodilation at each site. The infusion of L‐NAME persisted until a new, lower plateau in skin blood flow was achieved (post‐L‐NAME plateau; approximately 20–30 min of L‐NAME infusion) at which point maximal vasodilation was elicited by heating each site to 43°C and infusing each microdialysis fiber with 54 mM sodium nitroprusside.

### 2.5 Data acquisition and analysis

Data were recorded at 40 Hz using commercially available hardware and software (Powerlab 16/35 and Lab Chart 8, ADInstruments, Colorado Springs, CO) and saved to the hard drive of a personal computer (iMac, Apple, Cuppertino, CA). The following components of the laser‐Doppler recording were analyzed: (Tsao et al., 2023) baseline was averaged over the 3 min prior to the local heating protocol, (2) local heating plateau was averaged over the 3 min prior to infusion of L‐NAME, (3) post‐L‐NAME plateau was averaged over the 3 min prior to eliciting maximal vasodilation, and (4) maximal vasodilation was averaged over the final 3 min of the experimental protocol. Cutaneous vascular conductance (CVC) was calculated as laser‐Doppler flow divided by mean arterial pressure and normalized to maximal vasodilation (% CVC_max_). Percent NO‐dependent (% NO) vasodilation at each microdialysis site was calculated as: [(local heating plateau−post‐L‐NAME plateau) ÷ lSocal heating plateau] × 100 (Minson et al., 2001; Wong, 2013).

### 2.6 Statistical analysis

Power analysis was conducted, a priori, based on pilot data from our lab. Using the smallest difference in %NO between control (42 ± 13% NO) and tempol (57 ± 12% NO) sites in non‐Hispanic Black participants. Based on an effect size of 1.2, α of 0.05, and 80% power, a sample size of 12 participants was required. To account for potential differences in mean and standard deviation between pilot data and data from the sample population, we increased our sample size by 25%, which resulted in a final sample size of 15 participants. The skin blood flow components described above were analyzed using a general linear model (i.e., two‐way analysis of variance) with factors of group (non‐Hispanic Black and White) and microdialysis site (control, tempol, 1400 W, tempol + 1400 W). Tukey's post hoc test was used to account for multiple pairwise comparisons. The *p* values do not provide information as to the magnitude of observed differences and statistical guidelines are to not report *p* values alone (Wasserstein & Lazar, 2016). Therefore, data are shown as mean ± standard deviation with 95% confidence intervals of the difference (CI; lower limit, upper limit) and effect sizes (*d*). All data were compared and graphed using commercially available software (SAS, Cary, NC; Prism 9, GraphPad Software, Boston, MA). The level of significance was set at *p* ≤ 0.05.

## 3 RESULTS

**FIGURE 1:**
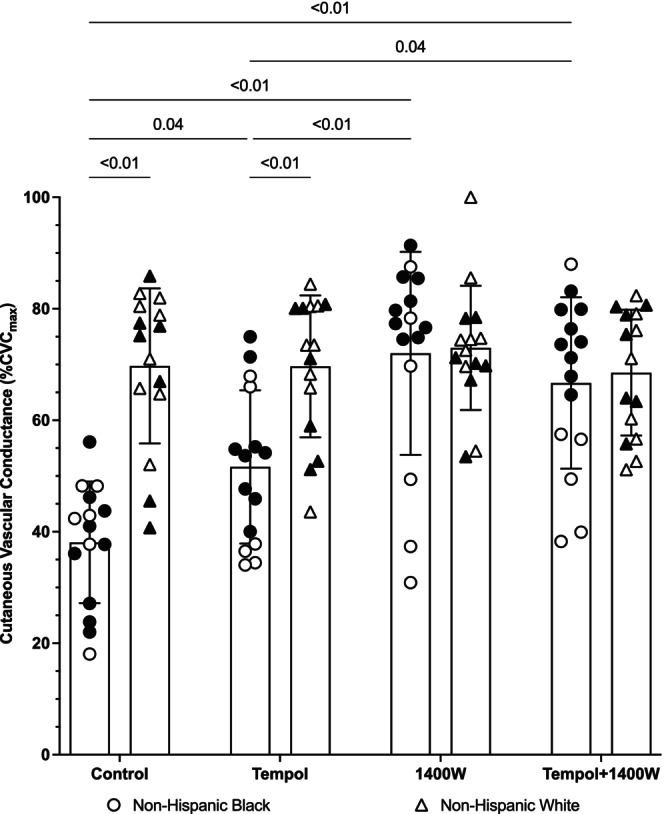
Group Microvascular endothelial function in non‐Hispanic Black (*n* = 15) and White (*n* = 15) young adults. Microvascular endothelial function was assessed as the plateau phase in response to local heating. The plateau was reduced in non‐Hispanic Black participants (circles) relative to non‐Hispanic White participants (triangles) at control sites (*p* < 0.01). In non‐Hispanic Black participants, tempol increased the plateau relative to control (*p* = 0.04, *d* = 1.09) but the plateau at tempol sites was still reduced relative to the tempol site in non‐Hispanic White participants (*p* < 0.01). Compared to control, there was a significant increase in the plateau in non‐Hispanic Black participants at both the 1400 W sites and the tempol +1400 W sites (both *p* < 0.01). The plateau at 1400 W (*p* < 0.01) and tempol +1400 W (*p* = 0.04, *d* = 1.03) was greater than at tempol sites in non‐Hispanic Black participants. The magnitude of the increase at 1400 W (*p* = 0.86) and tempol + 1400 W (*p* = 0.71) sites was such that the plateau in non‐Hispanic Black participants was not different than the plateau in non‐Hispanic White participants. Data for women in each group are shown in white symbols and data for men in black symbols. Data were analyzed using a general linear model with factors of group x microdialysis site. There was a significant main effect for group (*p* < 0.01) and microdialysis site (*p* < 0.01) and a significant interaction effect (*p* < 0.01). Data are shown as mean ± standard deviation (confidence intervals and effect sizes are in the main text). For clarity, only *p* ≤ 0.05 are shown on the graph.

**FIGURE 2:**
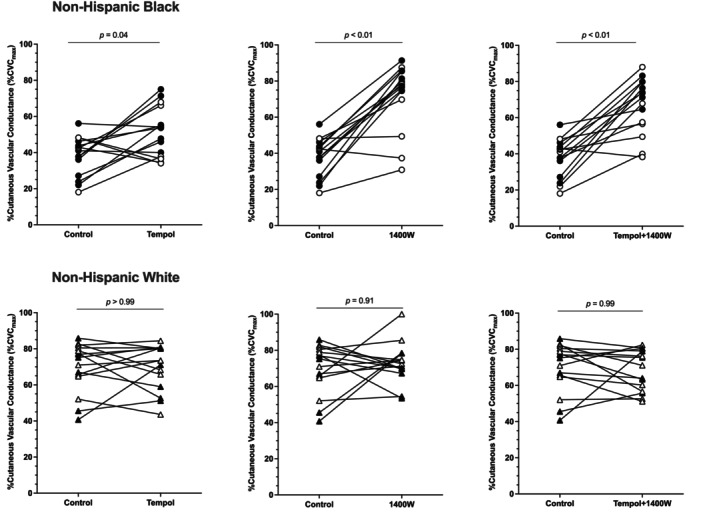
Individual plateau responses to each microdialysis treatment in non‐Hispanic Black (*n* = 15) and non‐Hispanic White (*n* = 15) young adults. The plateau response to each microdialysis treatment is shown for each participant. Data for non‐Hispanic Black participants (top three panels) are shown as circles and data from non‐Hispanic White participants (bottom three panels) are shown as triangles. Data for women in each group are shown in white symbols and data for men in each group are shown in black symbols. See Figure 1 and main text for means, standard deviations, and additional pairwise comparisons.

For transparency, data from women are shown in black symbols and data from men in white symbols in Figures 1 and 2.

### 3.1 Baseline, Post‐L‐NAME plateau, and maximal data (Table 3)

**TABLE 3:** Baseline, Post‐L‐NAME plateau, and maximal data.

	non‐Hispanic black	non‐Hispanic white
Baseline, % CVC_max_		
Control	12 ± 4	12 ± 4
Tempol	13 ± 7	14 ± 5
1400 W	10 ± 4	11 ± 3
Tempol + 1400 W	11 ± 3	13 ± 4
Post‐L‐NAME plateau, % CVC_max_		
Control	21 ± 6* (4, 12)	13 ± 5
Tempol	18 ± 7	14 ± 5
1400 W	14 ± 6** (2, 12)	14 ± 6
Tempol + 1400 W	12 ± 7** (4, 15)	16 ± 7
Maximal CVC, a.u./mmHg		
Control	1.94 ± 0.36	1.93 ± 1.15
Tempol	1.97 ± 0.48	1.81 ± 0.89
1400 W	2.21 ± 0.61	1.73 ± 0.60
Tempol + 1400 W	2.24 ± 0.59	1.79 ± 0.91

*Note*: Data are shown as mean ± SD (95% CI of the difference; lower limit, upper limit for statistically significant *p* values). CVC, cutaneous vascular conductance, calculated as laser‐Doppler flux (a.u.) divided by mean arterial pressure; %CVC_max_, percent maximal cutaneous vascular conductance; a.u., arbitrary units. *, *p* < 0.05 vs. non‐Hispanic White participants at same site; **, *p* < 0.05 vs. control site within the same group.

There was no main effect for group (*p* = 0.24) or microdialysis site (*p* = 0.11) or interaction effects (*p* = 0.62) for baseline % CVC_max_. Similarly, there was no main effect for group (*p* = 0.23) or microdialysis site (*p* = 0.77) or interaction effects (*p* = 0.17) for maximal CVC.

For post‐L‐NAME plateau % CVC_max_, there was a significant group x microdialysis site interaction effect (*p* < 0.01). The post‐L‐NAME plateau was significantly higher in non‐Hispanic Black participants relative to non‐Hispanic White participants at control sites (*p* < 0.01; *d* = 1.45). Within the non‐Hispanic Black participant cohort, the post‐L‐LNAME plateau was reduced at 1400 W (*p* < 0.01; *d* = 1.17) and tempol +1400 W (*p* < 0.01; *d* = 1.37) sites compared to control but there were no other differences between sites. There were no differences within the non‐Hispanic White participant cohort.

### 3.2 Local heating plateau

The group data for the plateau are shown in Figure 1. The individual responses to each microdialysis treatment are shown in Figure 2. There was a significant group x microdialysis site interaction effect for the local heating plateau (*p* < 0.01). At control sites, the plateau in non‐Hispanic Black participants was 38 ± 11% CVC_max_ and 70 ± 14% CVC_max_ in non‐Hispanic White participants, which was statistically significant (*p* < 0.01; *d* = 2.54; 95% CI: −41, −22). At tempol sites, the plateau was 52 ± 14% CVC_max_ and 70 ± 13% CVC_max_ in non‐Hispanic Black and White participants, respectively (*p* < 0.01; *d* = 1.33; 95% CI: −28, −8). The plateau at 1400 W sites in non‐Hispanic Black participants was 72 ± 18% CVC_max_ and 73 ± 11% CVC_max_ in non‐Hispanic White participants. There was no difference between groups at 1400 W sites (*p* = 0.86; *d* = 0.07; 95% CI: −12, 10). At tempol + 1400 W sites, the plateau was 65 ± 18 % CVC_max_ in non‐Hispanic Black participants and 69 ± 11%CVC_max_ in non‐Hispanic White participants; there was no statistical difference between groups (*p* = 0.57; *d* = 0.27; 95% CI: −12, 8).

Within the non‐Hispanic Black participant cohort, the plateau at tempol (*p* = 0.04; *d* = 1.09; 95% CI: −0.3, −27), 1400 W (*p* < 0.01; *d* = 2.16; 95% CI: −20, −48), and tempol + 1400 W (*p* < 0.01; *d* = 1.72; 95% CI: −15, −42) sites was increased compared to control. The plateau at 1400 W (*p* < 0.01; *d* = 1.22; 95% CI: −5, −36) and tempol + 1400 W (*p* = 0.04; *d* = 0.79; 95% CI: −1, −30) sites was significantly increased compared to tempol sites. There was no difference between 1400 W and tempol + 1400 W sites (*p* = 0.63; *d* = 0.39; 95% CI: −8, 18). There were no differences within the non‐Hispanic White participant cohort (all *p* values >0.71; range of *d* = 0–0.23).

### 3.3 Nitric oxide‐dependent vasodilation

**FIGURE 3:**
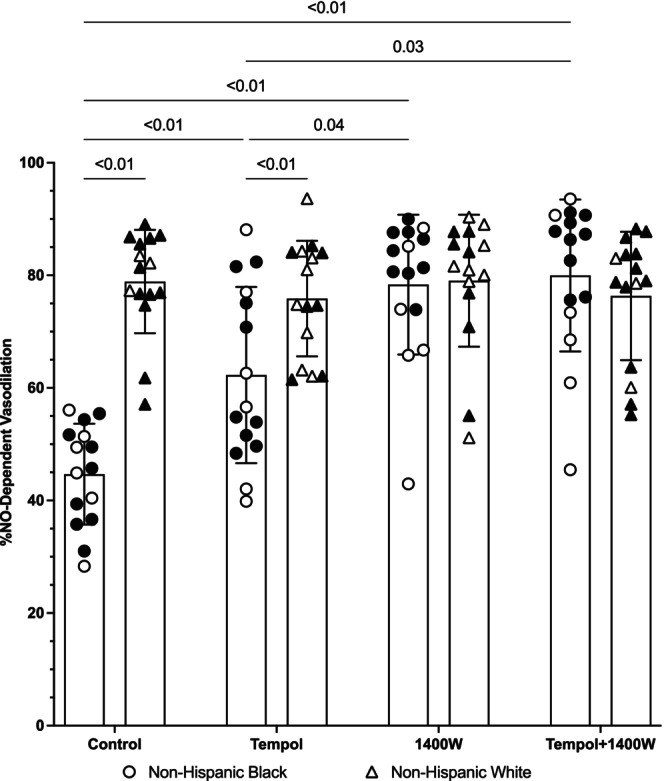
Calculated NO‐dependent vasodilation in non‐Hispanic Black (*n* = 15) and White (*n* = 15) young adults. NO‐dependent vasodilation was reduced in non‐Hispanic Black participants (circles) relative to non‐Hispanic White participants (triangles) at control sites (both *p* < 0.01). In non‐Hispanic Black participants, tempol increased NO‐dependent vasodilation relative to control (*p* < 0.01) but this was still reduced relative to the tempol site in non‐Hispanic White participants (*p* < 0.01). Compared to control, there was a significant increase in NO‐dependent vasodilation in non‐Hispanic Black participants at both the 1400 W sites and the tempol +1400 W sites (both *p* < 0.01). NO‐dependent vasodilation at 1400 W (*p* = 0.04, *d* = 1.1) and tempol +1400 W (*p* = 0.03, *d* = 1.22) sites was also greater than at tempol sites in non‐Hispanic Black participants. The magnitude of the increase at 1400 W (*p* = 0.88) and tempol +1400 W (*p* = 0.33) sites was such that the plateau in non‐Hispanic Black participants was not different than the plateau in non‐Hispanic White participants. Data for women in each group are shown in white symbols and data for men in black symbols. Data were analyzed using a general linear model with factors of group x microdialysis site. There was a significant main effect for group (*p* < 0.01) and microdialysis site (*p* < 0.01) and a significant interaction effect (*p* < 0.01). Data are shown as mean ± standard deviation (confidence intervals and effect sizes are in the main text). For clarity, only *p* ≤ 0.05 are shown on the graph.

**FIGURE 4:**
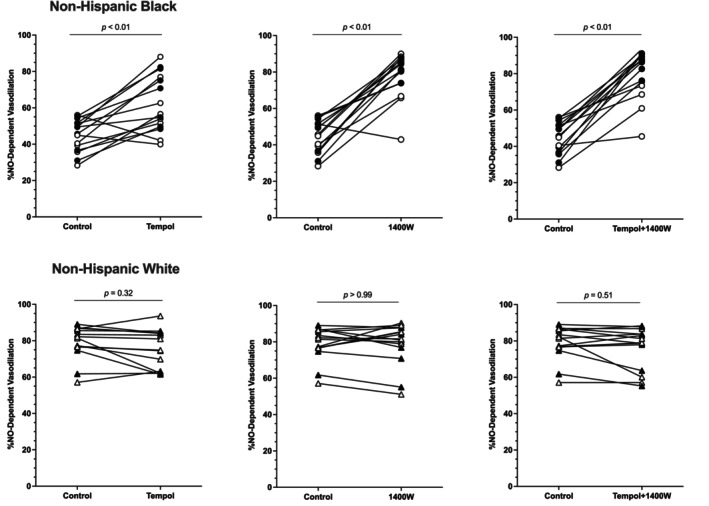
Individual NO‐dependent vasodilation to each microdialysis treatment in non‐Hispanic Black (*n* = 15) and non‐Hispanic White (*n* = 15) young adults. The calculated NO‐dependent vasodilation to each microdialysis treatment is shown for each participant. Data are shown as the response from control to each treatment site. Data for non‐Hispanic Black participants (top three panels) are shown as circles and data from non‐Hispanic White participants (bottom three panels) are shown as triangles. Data for women in each group are shown in white symbols and data for men in each group are shown in black symbols. See Figure 3 and main text for means, standard deviations, and additional pairwise comparisons.

The group data for NO‐dependent vasodilation are shown in Figure 3. The individual responses to each microdialysis treatment are shown in Figure 4. There was a significant group x microdialysis site interaction effect for NO‐dependent vasodilation (*p* < 0.01). At control sites, NO‐dependent vasodilation was 45 ± 9% NO for non‐Hispanic Black participants and 79 ± 9%NO for non‐Hispanic White participants and this was statistically significant between groups (*p* < 0.01; *d* = 3.78; 95% CI: −41, −27). At tempol sites, NO‐dependent vasodilation for non‐Hispanic Black participants was 62 ± 16% NO and 76 ± 10% NO for non‐Hispanic White participants. There was a statistically significant difference at tempol sites (*p* < 0.01; *d* = 1.05; 95% CI: −24, −4). At 1400 W sites, NO‐dependent vasodilation for non‐Hispanic Black participants was 78 ± 12% NO and 79 ± 12% NO for non‐Hispanic White participants; there was no difference between groups (*p* = 0.88; *d* = 0.08; 95% CI: −10, 8). At tempol + 1400 W, NO‐dependent vasodilation was 80 ± 13% NO for non‐Hispanic Black participants and 75 ± 15% NO for non‐Hispanic White participants; there was no statistical difference between groups (*p* = 0.43; *d* = 0.37; 95% CI: −6, 13).

Within the non‐Hispanic Black participant cohort, there was a significant increase in %NO at tempol (*d* = 1.22; 95% CI: −7, −29), 1400 W (*d* = 3.05; 95% CI: −22, −46), and tempol +1400 W (*d* = 3.03; 95% CI: −24, −46) sites compared to control sites (all *p* < 0.01). Compared to tempol sites, % NO was significantly greater at 1400 W (*p* = 0.04, *d* = 1.11; 95% CI: −1, −32) and tempol +1400 W (*p* = 0.03; *d* = 1.22; 95% CI: −2, −34) sites. There was no difference between 1400 W and tempol +1400 W sites (*p* = 0.95; *d* = 0.16; 95% CI: −10, 7). There were no differences within the non‐Hispanic White participant cohort (all *p* > 0.21; range *d* = 0–0.31).

## 4 DISCUSSION

The main finding from the present study is that superoxide and iNOS are partly responsible for the lower endothelial‐dependent and NO‐dependent microvascular vasodilation in non‐Hispanic Black young adults compared with non‐Hispanic White young adults. These results are supported by the observation that inhibition of superoxide (tempol) and iNOS (1400 W) both increase the plateau of local heating (an index of generalized endothelial microvascular function) as well as the contribution of NO to the cutaneous microvascular response to local heating (Figures 1, 2, 3, 4). While inhibition of superoxide and iNOS both augment cutaneous microvascular function, the increases observed with iNOS inhibition appear to be more potent than the effects of superoxide, suggesting inhibition of iNOS may be a more effective therapeutic target for augmenting microvascular function. Moreover, since combined tempol + 1400 W had no greater effect than 1400 W alone, these data also suggest there is no additional benefit of simultaneously inhibiting superoxide and iNOS.

The plateau phase of the local heating response is largely mediated by NO derived from eNOS (~60%–80%) (Bruning et al., 2012; Choi et al., 2014; Kellogg Jr., Liu, et al., 1999; Kellogg Jr. et al., 2008, 2009; Minson et al., 2001). Consistent with data from our lab and others (Akins et al., 2022; Hurr et al., 2018; Kim et al., 2018; Kim & Brothers, 2020; Miller et al., 2021; Patik et al., 2018; Turner, Hayat, et al., 2023; Wolf et al., 2020; Wong et al., 2020), we found there is both reduced microvascular endothelial‐dependent vasodilation (plateau; Figures 1 and 2) and reduced NO‐dependent vasodilation (Figures 3 and 4) to a standardized local heating protocol in non‐Hispanic Black young adults. Reduced microvascular endothelial function and NO‐dependent vasodilation is observed whether local heating to 39°C is used, as in the current study (Akins et al., 2022; Hurr et al., 2018; Kim & Brothers, 2020; Miller et al., 2021; Patik et al., 2018; Turner, Hayat, et al., 2023; Wong et al., 2020), or to 42°C (Kim et al., 2018).

The increase in microvascular endothelial function and NO‐dependent vasodilation at tempol compared to control sites suggests superoxide negatively affects the microvascular endothelium in non‐Hispanic Black young adults. The increase in the plateau with tempol is consistent with previous data (Hurr et al., 2018) but contrasts with our recent publication where we failed to observe an effect of tempol (Turner, Hayat, et al., 2023). While our results for the plateau differed, our data showing improved NO‐dependent vasodilation was remarkably similar between our two studies (Turner, Hayat, et al., 2023). The reason for the differing plateau response to tempol between our two studies is unclear. In both studies, we used the same concentration of tempol. We also included men and women in both studies but did not control for menstrual cycle/oral contraceptive phase. Recent data suggests inhibition of oxidative stress has greater effects in non‐Hispanic Black men compared to non‐Hispanic Black women (Patik et al., 2018), but women were only tested during the low hormone phase of the menstrual/contraceptive pill phase. In the present study, two women were tested during the active pill phase of contraceptive use and four women declined to disclose this information so it's possible our current observations are at least partially influenced by testing women during different phases of the menstrual/contraceptive pill cycle. The plateau phase does appear to be highly variable relative to the magnitude of the response in both non‐Hispanic Black and White participants. In comparison, the calculated NO‐dependent component has a larger absolute value than the plateau but with similar, or only slightly larger, measures of variability. It is therefore plausible that the differing statistical outcomes are more a mathematical outcome than a physiological outcome. We did not calculate the effect size in our previous publication; however, calculation of the effect size from the reported data indicates a large effect size of *d* = 1.10, which is comparable to *d* = 1.09 in the present study (Turner, Hayat, et al., 2023). Based on these large effect sizes, it appears tempol is having physiological effects that are not necessarily evident from *p* values alone. Regardless of the reason(s), superoxide does appear to have a detrimental effect on the microvasculature in non‐Hispanic Black young adults and these detrimental effects can be mitigated with tempol (Hurr et al., 2018).

The effect of iNOS on cutaneous microvascular function in non‐Hispanic Black young adults is more consistent with our previous data (Miller et al., 2021). In both a previous publication and in the current study, we show that iNOS makes a large contribution to the reduced microvascular endothelial function and NO‐dependent vasodilation in non‐Hispanic Black young adults (Miller et al., 2021). Upregulation of iNOS typically occurs under conditions of stress such as heightened nitrosative and/or oxidative stress. The upregulation of iNOS subsequently results in an overproduction of NO, which exacerbates the stress response. In our previous study (Miller et al., 2021), we did not inhibit oxidative stress, and, as such, were unable to address whether there were any interactions between iNOS and oxidative stress. The current data indicate concomitant inhibition of superoxide and iNOS does not yield any additional benefit than iNOS inhibition alone on either the plateau or the calculated NO‐dependent vasodilation. That is, the increase in CVC at tempol +1400 W sites was not any greater than that observed at 1400 W only sites (Figures 1, 2, 3, 4). Both the 1400 W site and the tempol + 1400 W site were found to be greater than the tempol site for both the plateau and NO‐dependent vasodilation. This was supported not only statistically but also based on the range of effect sizes of *d* = 1.03–1.22, indicating significant physiological differences in the effects of tempol compared to 1400 W. Mechanistically, it's possible 1400 W reduces oxidative stress, nitrosative stress, and/or proinflammatory cytokines while also directly suppressing the activity of iNOS (Massey et al., 2023; Méndez‐Valdés et al., 2023; Putra et al., 2020; Tsai & Lein, 2021). Conversely, tempol may only be exerting an antioxidant effect with no direct action on iNOS. The pleiotropic effects of 1400 W may explain why inhibition of iNOS yields greater improvements in both microvascular endothelial function as well as eNOS‐mediated NO‐dependent vasodilation compared to inhibition of superoxide alone.

### 4.1 Experimental considerations

There are several experimental considerations that may affect data interpretation. First, we did not address social determinants of health but recognize the importance social determinants of health can have on physiological outcomes. A recent study found no correlation between socioeconomic status and cutaneous microvascular function (Wolf et al., 2020), but it is possible the study was underpowered for questionnaire‐based outcomes. Second, to increase external validity, we did not control for menstrual cycle or oral contraceptive phase (Stanhewicz & Wong, 2020). We have previously shown that menstrual cycle/oral contraceptive phase can have different effects on cutaneous microvascular responses to local heating (Turner, Stanhewicz, et al., 2023). Another study has shown that inhibition of oxidative stress (specifically, NADPH oxidase and xanthine oxidase) is most effective in non‐Hispanic Black men with minimal to no effect in women (women were tested during low hormone phases) (Patik et al., 2018). Most of the women in this study were tested during low hormone phases and most of the women showed improvements with tempol (open symbols in Figures 1, 2, 3, 4). Nevertheless, we cannot discount the possibility that the pharmacological effects may have different effects depending on menstrual cycle/oral contraceptive phase or that there may be different effects between the natural menstrual cycle and oral contraceptives. Because we did not measure sex hormones in women or men, we are unable to directly determine if there are any interactions among sex hormone concentrations or any of our outcome variables. We also had four women choose not to disclose their cycle information due to privacy concerns and we did not to exclude these participants from the study. Third, we did not analyze blood samples for biomarkers of oxidative stress, nitrosative stress, inflammation, etc. We have previously shown variable outcomes to these measures despite improved microvascular responses to iNOS inhibition (Miller et al., 2021), but we recognize that we cannot directly assert whether oxidative or nitrosative stress contributed to the current findings.

### 4.2 Conclusion

We found that independent inhibition of superoxide and iNOS both increase measures of microvascular function in non‐Hispanic Black young adults. Inhibition of iNOS appeared to impart greater improvements in microvascular function than inhibition of superoxide while concomitant inhibition of superoxide and iNOS conferred no greater benefit than inhibition of iNOS alone. Collectively, these data suggest inhibition of iNOS may be an important target for improving microvascular function and, potentially, cardiovascular health in this population.

## CONFLICT OF INTEREST STATEMENT

The authors have no conflict of interest to disclose.

## FUNDING INFORMATION

This study was funded by NIH grant R01 HL141205 to Brett Wong.

## ETHICS STATEMENT

All experimental procedures were initially approved by Advarra Institutional Review Board (Columbia, MD; Pro00024265) and accepted by the Georgia State University Institutional Review Board, and approval for the use of all pharmacological substances was provided by the U.S. Food and Drug Administration (IND 138231). This study conformed to the guidelines set forth by the Declaration of Helsinki. All participants provided written and verbal informed consent prior to participating in any experimental procedure.

## Data Availability

The data that support the findings of this study are available from the corresponding author upon reasonable request.
